# Interpretation of *SLC3A1* and *SLC7A9* variants in cystinuria patients: The significance of the PM3 criterion and protein stability

**DOI:** 10.1007/s00240-023-01466-y

**Published:** 2023-07-13

**Authors:** Beomki Lee, Soo-Youn Lee, Deok Hyun Han, Hyung-Doo Park

**Affiliations:** 1 Department of Laboratory Medicine and Genetics, Samsung Medical Center, Sungkyunkwan University School of Medicine, 81 Irwon-ro, Gangnam-gu, Seoul, 06351 Korea; 2 Department of Urology, Samsung Medical Center, Sungkyunkwan University School of Medicine, 81 Irwon-ro, Gangnam-gu, Seoul, 06351 Korea; 3 Department of Medical Device Management and Research, SAIHST, Sungkyunkwan University, Seoul, 06355 Korea; 4 Present Address: Graduate School of Medical Science and Engineering, Korea Advanced Institute of Science and Technology (KAIST), Daejeon, Korea

**Keywords:** Cystinuria, *SLC3A1*, *SLC7A9*, Genotype, Phenotype, Penetrance, Variant interpretation, Protein stability

## Abstract

Cystinuria is a genetic disorder caused by defects in the b^0,+^ transporter system, which is composed of rBAT and b^0,+^AT coded by *SLC3A1* and *SLC7A9*, respectively. Variants in *SLC3A1* and *SLC7A9* follow autosomal recessive inheritance and autosomal dominant inheritance with reduced penetrance, respectively, which complicates the interpretation of cystinuria-related variants. Here, we report seven different *SLC3A1* variants and six different *SLC7A9* variants. Among these variants were two novel variants previously not reported: *SLC3A1* c.223C > T and *SLC7A9* c.404A > G. In silico analysis using REVEL correlated well with the functional loss upon *SLC7A9* variants with scores of 0.8560–0.9200 and 0.4970–0.5239 for severe and mild decrease in transport activity, respectively. In addition, DynaMut2 was able to predict a decreased protein expression level resulting from the *SLC7A9* variant c.313G > A with a ΔΔG^Stability^ −2.93 kcal/mol. Our study adds to the literature as additional cases of a variant allow applying the PM3 criterion with higher strength level. In addition, we suggest the clinical utility of REVEL and DynaMut2 in interpreting *SLC3A1* and *SLC7A9* variants. While a decreased protein expression level is not embraced in the current variant interpretation guidelines, we believe in silico protein stability predicting tools could serve as evidence of protein function loss.

## Introduction

Cystinuria (OMIM: 220,100) is a genetic disorder caused by defects in the dibasic amino acid transporters, which results in elevated urinary cystine along with dibasic amino acids such as arginine, ornithine, and lysine [1]. Cystinuria has traditionally been classified into three categories based on the level of urinary amino acid excretion. Following identification of the underlying genetic cause, the disorder is now labeled type A and B for variants in *SLC3A1* (OMIM: 104,614) and *SLC7A9* (OMIM: 604,144), respectively [2].

The b^0,+^ system is composed of rBAT and b^0,+^AT coded by *SLC3A1* and *SLC7A9*, respectively, which form a heterodimer with a disulfide bond [3, 4]. While the b^0,+^AT subunit transports the amino acids, the rBAT subunit plays a role in trafficking and maturation of the complex [4–6]. The inheritance pattern of cystinuria-related variants also shows a distinct pattern. Variants in *SLC3A1* are inherited in an autosomal recessive order, whereas those in *SLC7A9* follow an autosomal dominant with incomplete penetrance pattern. This paradigm seems obvious considering the function of proteins encoded by each gene per se.

While a number of variants have been discovered as a cause of cystinuria, interpretation of variants in *SLC3A1* and *SLC7A9* is still challenging. First, the inheritance pattern complicates the interpretation. In the autosomal dominant with incomplete penetrance pattern, it is difficult to determine the pathogenicity when there are two variants of uncertain significance in one gene as either or both variants could contribute to the disease. Second, genetic heterogeneity of cystinuria is another concern. When there are variants of question in both *SLC3A1* and *SLC7A9*, it is not straightforward to ascertain which variant is causative. Moreover, there are cystinuria patients without any pathogenic variants in *SLC3A1* and *SLC7A9*. As it is currently suspected there could be other genes associated with cystinuria [7], relating a novel variant in *SLC3A1* and/or *SLC7A9* with cystinuria is challenging.

In this study, we report ten cystinuria cases, which include two novel variants. We believe the current study will serve as evidence in interpreting *SLC3A1* and *SLC7A9* variants. Furthermore, while protein stability is an attribute not employed in the current guidelines for variant interpretation, we have evaluated protein stability change caused by variants to unveil its potential utility in determining the pathogenicity of a given variant.

## Methods

### Study population

As the aim of this study was to investigate genetic variants in cystinuria patients, those with *SLC3A1* and *SLC7A9* sequencing orders were included. Additional clinical information of the enrolled cystinuria patients were obtained, which included age of onset, familial history of urolithiasis, clinical manifestation, intervention, chemical composition of the urinary stone, urine amino acid levels, and identified genetic variants of *SLC3A1* and *SLC7A9* genes. This study was approved by the Institutional Review Board (IRB) of Samsung Medical Center (IRB No. 2022–05-064), and the need for written informed consent was wived due to the anonymous and retrospective nature of this study.

### Laboratory analyses

Urine amino acid levels were quantitatively measured with liquid chromatography tandem mass spectrometry (LC–MS/MS) to reveal changes in urinary dibasic amino acid levels including cystine. After urologic intervention of each patient, urinary stone analysis was performed to examine the chemical composition through Fourier transform infrared spectroscopy (FT-IR) using the FT-IR system 2000 (PerkinElmer, Wallac Oy, Turku, Finland) and Spectrum software (PerkinElmer) described in a previous publication [8]. For genetic analysis, DNA was extracted from whole blood using a Roche MagNA Pure 96 DNA isolation kit (Roche Applied Science, Manheim, Germany). The *SLC3A1* and *SLC7A9* gene sequences were obtained with polymerase chain reaction (PCR) and full sequencing using an ABI Prism 3730XL DNA sequencer (Applied Biosystems, Foster City, CA, USA). The in-house designed primers are available upon reasonable request. Nucleotides were numbered according to the transcript sequences of *SLC3A1* (NM_000341.3) and *SLC7A9* (NM_014270.4).

### Evaluation of *SLC7A9* variants with known functional changes

As there was one previous study demonstrating the functional change in *SLC7A9* variants experimentally [9], we evaluated the feasibility of using the pathogenicity score of REVEL [10] and predicted stability change (ΔΔG^Stability^) calculated with DynaMut2 [11].

### Variant interpretation

The identified variants were interpreted applying the ACGS Best Practice Guidelines for Variant Classification 2019 [12], which is based on the 2015 ACMG/AMP guidelines [13]. In addition, variants with one very strong criterion along with one supporting criterion were regarded as likely pathogenic according to the ClinGen Sequence Variant Interpretation (SVI) Recommendation for PM2 [14]. The PM3 criterion was applied following the guidance of ClinGen SVI recommendation, which allows for applying different strength levels based on the phasing of two variants and classification of the variant other than the variant of interest [15]. The PM1_Supporting criterion was assigned if a variant was predicted to affect protein function according to Martell et al. (2017) [16]. For investigation of previous literature of a certain variant, HGMD Professional (2022.1) [17] and Mastermind [18] were utilized. To analyze the population frequency, gnomAD v2.1.1 [19] and KRGDB_1722 [20] were used. For in silico prediction, REVEL [10] and SpliceAI [21] were utilized for single nucleotide variants and splice site variants, respectively. Predicted stability change (ΔΔG^Stability^) was calculated with DynaMut2 [11]. For novel missense variants, the structure of the resulting protein was illustrated using Missense3D [22].

### Using DynaMut2 to predict protein instability

DynaMut2 is an in silico tool designed to predict a change in protein stability upon missense variants [11]. The authors of the DynaMut2 claim its usefulness in predicting the role of variants in disease [11]. The predicted stability change (ΔΔG^Stability^) values were obtained with DynaMut2 where negative values indicate a destabilizing effect and positive values indicate a stabilizing effect. The greater the absolute value of ΔΔG^Stability^, the greater the effect. The ΔΔG^Stability^ values were classified into seven categories: highly destabilizing (ΔΔG^Stability^ < −1.84 kcal/mol), destabilizing (−1.84 kcal/mol < ΔΔG^Stability^ < −0.92 kcal/mol), slightly destabilizing (−0.92 kcal/mol < ΔΔG^Stability^ < −0.46 kcal/mol), neutral (−0.46 kcal/mol < ΔΔG^Stability^ < 0.46 kcal/mol), slightly stabilizing (0.46 kcal/mol < ΔΔG^Stability^ < 0.92 kcal/mol), stabilizing (0.92 kcal/mol < ΔΔG^Stability^ < 1.84 kcal/mol), and highly stabilizing (ΔΔG^Stability^ > 1.84 kcal/mol). The PDB accession number 6LID [23] was used as the reference structure of the b^0,+^AT-rBAT complex. Since the b^0,+^AT-rBAT complex is a dimer of heterodimers consisting of b^0,+^AT and rBAT, the ΔΔG^Stability^ both when one dimer was affected and when both dimers were affected were calculated.

## Results

### Clinical characteristics

The clinical characteristics of the cystinuria patients are summarized in Table 1. While all patients had multiple events of urolithiasis, only two patients had a familial history of urolithiasis. The identified variants, stone component, and urine amino acid levels are listed in Table 2. Stones from all patients consisted of 100% cystine. Increased urinary cystine and dibasic amino acid levels were noted in all patients that were tested.

**Table 1:** Clinical characteristics of cystinuria patients

Case	Gender	Onset age	FHx	Initial presentation	Recurrent urolithiasis	Urological interventions
1	M	37 years	+	Stone (bilateral staghorn)	+	Both PCNL
2	M	22 years	–	Stone (spontaneous passage)	+	Lt. ESWL, Rt. PCNL, Lt. UL, Rt. UL
3	M	24 years	–	Stone (spontaneous passage)	+	Lt. PCNL, Lt. UL
4–1	F	20 years	+	Stone (spontaneous passage)	+	Rt. PCNL
4–2	F	14 years	+	Stone (Rt. ureter)	+	Rt. UL, Rt. ESWL, Lt. UL
5	M	10 months	–	Stone (spontaneous passage)	+	OU, Rt. UL, Lt. UL, Rt. ESWL, Lt. ESWL, Lt. PCNL
6	F	6 years	–	Stone (Rt. staghorn)	+	Rt. PNL, Rt. ESWL, Rt. UL
7	M	32 years	–	Stone (Lt. renal)	+	Lt. ESWL, Rt. UL
8	M	34 years	NA	Stone (Rt. renal)	+	Rt. OU, Lt. ESWL, Lt. UL, Lt. OU
9	M	11 years	–	Stone (Rt. renal)	+	Rt. OU, Lt. PCNL, Rt. PCNL, Rt. ESWL

*FHx* family history, *CKD* chronic kidney disease, *NA* not available, *ESWL* extracorporeal shockwave lithotripsy, *PCNL* percutaneous nephrolithotomy, *UL* ureterorenoscopic lithotomy, *OU* open urolithotomy

**Table 2:** Identified variants, urinary stone composition, and urine amino acid levels of each patient

Case	*SLC3A1*	*SLC7A9*	Genotype	Stone analysis	Urine amino acid (μmol/g Cr)				
					Cys	Orn	Lys	Arg	Sum
1	ND	c.1305G > A (Het)	B-	Cystine 100%	723	692	3815	359	5589
2	ND	c.404A > G(;)988G > A	B- or BB	Cystine 100%	1673	1536	8120	2007	13,336
3	c.223C > T(;)1318 T > C	ND	AA	Cystine 100%	1531	1380	3490	2546	8947
4–1	ND	c.1445C > T (Hom)	BB	Cystine 100%	2911	2131	8447	3216	16,705
4–2	ND	c.1445C > T (Hom)	BB	Cystine 100%	1155	2054	8492	2347	14,048
5	c.418G > A(;)1976A > C	ND	A- or AA	Cystine 100%	836	1515	5440	3486	11,277
6	ND	c.1060G > A(;)829G > A	B- or BB	Cystine 100%	NT	NT	NT	NT	NT
7	c.1500 + 1G > A (Hom)	NT	AA	Cystine 100%	2039	1600	6406	3143	13,188
8	c.1976A > C(;)2017 T > C	NT	A- or AA	Cystine 100%	2034	1289	6613	1327	11,263
9	c.1820del (Hom)	NT	AA	Cystine 100%	NT	NT	NT	NT	NT

*Het* heterozygous, *Hom* homozygous, *yrs* years, *Cys* cystine, *Orn* ornithine, *Lys* lysine, *Arg* arginine, *ND* not detected, *NT* not tested

### Evaluation of REVEL and DynaMut2 in predicting the degree of functional change

The REVEL score was able to distinguish variants with mild effect and severe effect in transport activity as all variants with a mild decrease in protein function had a REVEL score less than 0.6, while other variants with severe loss of function all had a REVEL score greater than 0.8. Among the evaluated variants, the p.(Gly105Arg) variant exhibited a significantly decreased protein expression by 10% of wild-type in transfected cells [9], which was also the only variant predicted to be highly destabilizing according to the DynaMut2 results with a ΔΔG^Stability^ of −2.93 kcal/mol when both SLC7A9 light chains of the dimer were composed of proteins with the p.(Gly105Arg) variant (Table 3). Variants other than p.(Gly105Arg) did not have a drastic effect on ΔΔG^Stability^, with the maximum effect being −1.65 kcal/mol by p.(Ala70Val) indicating a destabilizing effect.

**Table 3:** Predicted effects of protein change by multiple algorithms for variants with known functional change

Gene	Variant	Predicted effect	Transport activity^a^	REVEL	DynaMut2^b^
*SLC7A9*	c.209C > T	p.(Ala70Val)	Mild (78%)	0.5239	−0.37, −1.65
	c.313G > A	p.(Gly105Arg)^c^	Severe (10%)	0.9060	−0.79, −2.93
	c.508G > A	p.(Val170Met)	Severe (0%)	0.8750	−0.64, −0.67
	c.544G > A	p.(Ala182Thr)	Mild (60%)	0.4970	−0.30, −1.21
	c.997C > T	p.(Arg333Trp)	Severe (10%)	0.9200	0.16, 0.18
	c.1060G > A	p.(Ala354Thr)	Severe (0%)	0.8560	−0.70, −0.02

^a^The degree of functional change in each variant was adopted from Font et al. [9]

^b^The two values indicate the ΔΔG^Stability^ (kcal/mol) when one heterodimer is affected and when both chains are affected, respectively

^c^The amount of protein production was significantly decreased in transfected cells by 10% of wild-type according to Font et al. [9]

### Interpretation and classification of the identified variants

**Fig. 1:**
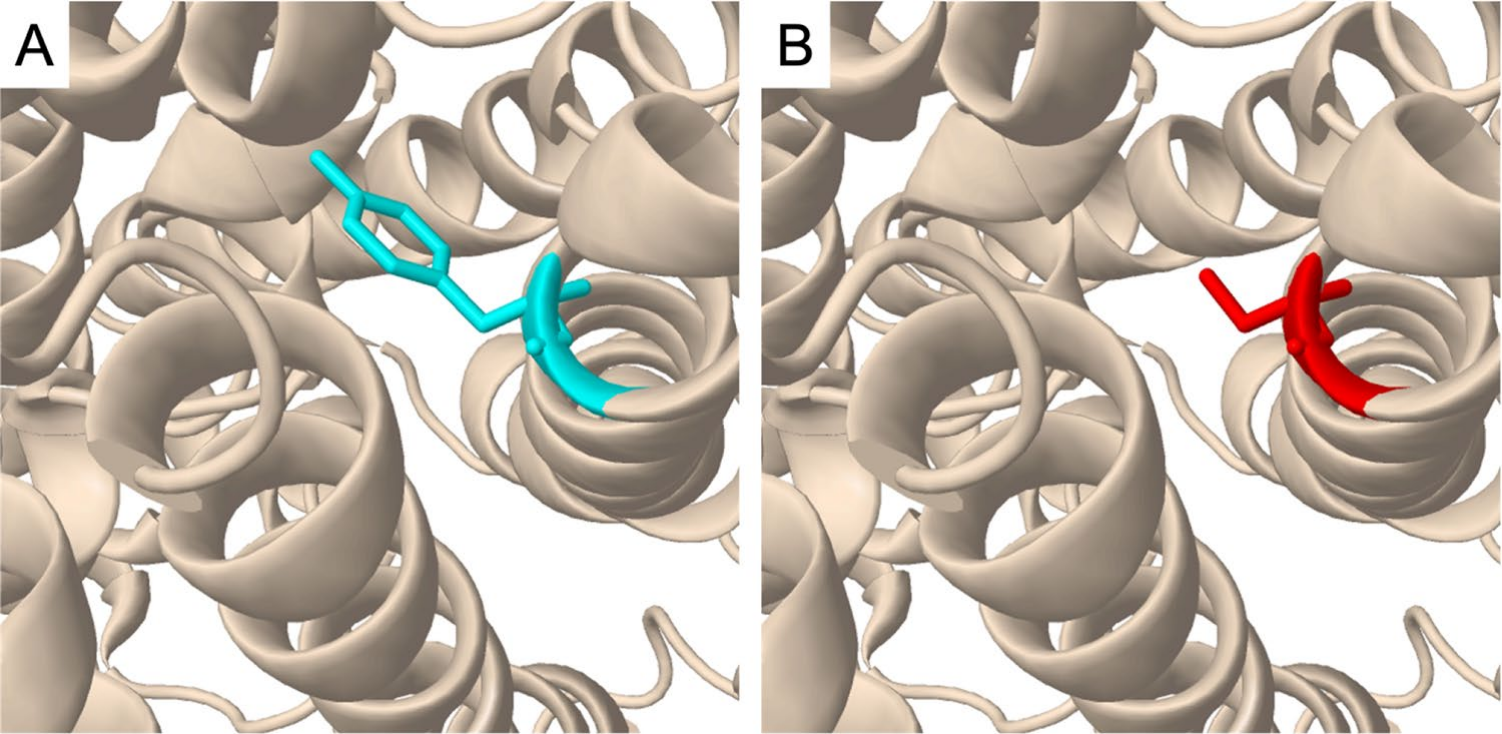
Structures of the **A** wild-type and **B** p.(Tyr135Cys) mutant SLC7A9 protein (the cyan color indicates the wild-type tyrosine residue and the red color indicates the mutant cysteine residue)

A total of 13 different variants were identified with seven different *SLC3A1* variants and six different *SLC7A9* variants including one novel *SLC3A1* variant and one novel *SLC7A9* variant not previously reported. Among the *SLC3A1* variants were three pathogenic variants, three likely pathogenic variants, and one variant of uncertain significance. Among the *SLC7A9* variants were one pathogenic variant, two likely pathogenic variants, and three variants of uncertain significance. While all variants identified in our study had an extremely low minor allele frequency in the gnomAD v2.1.1, NM_014270.4(SLC7A9):c.829G > A had a MAF of 0.6407% in KRGDB_1722. This variant also had the highest MAF in the gnomAD v2.1.1. among the variants identified in this study. In silico prediction of missense variants with REVEL resulted in (1) a score ≥ 0.8 for three *SLC3A1* variants and two *SLC7A9* variants, (2) 0.6 ≤ score < 0.8 for three *SLC7A9* variants, and (3) a score < 0.6 for one *SLC3A1* variant. SpliceAI predicted donor loss (score 1.00 at −1 bp) and gain (score 0.67 at 44 bp) in the c.1500 + 1G > A variant. All other variants identified in our patients were predicted to have no splicing effect according to SpliceAI. DynaMut2 predicted that all *SLC3A1* missense variants will cause a highly destabilizing effect when both chains of the dimer are affected and the p.(Tyr135Cys) variant of *SLC7A9* will cause a highly destabilizing effect even with one of the dimers affected. There were two novel variants identified: (1) NM_000341.3(SLC3A1):c.223C > T and (2) NM_014270.4(SLC7A9):c.404A > G. A novel pathogenic variant was identified in *SLC3A1*, whereas a novel variant of uncertain significance was identified from *SLC7A9*. Using Missense3D, it was predicted that the novel p.(Tyr135Cys) variant of *SLC7A9*, located at the end of the transmembrane helix, does not change the secondary structure of the protein (Fig. 1). In addition, the p.(Trp440Arg) variant of *SLC3A1* was interpreted as a likely pathogenic variant with the support of a case included in our study, which elevates the evidence level of the PM3 criterion. Refer to Table 4 for the list of variants, their interpretation, and related information; refer to Table 5 for details regarding the PM3 criterion. For further evaluation of the effects of compound heterozygote variants, the protein stability change affected by two variants was also predicted, which demonstrated that c.418G > A(;)1976A > C and c.1976A > C(;)2017 T > C would have highly destabilizing effects (Table 6).

**Table 4:** Interpretation of the variants identified in *SLC3A1* and *SLC7A9*

Gene	Variant	Predicted effect	Variant classification (evidence combinations)	gnomAD v2.1.1	KRGDB_1722	REVEL	DynaMut2^a^	References
*SLC3A1*	c.223C > T	p.(Gln75Ter)	PV (PVS1 + PM2 + PP4)	< 0.01%	0%	NA	NA	Novel
	c.418G > A	p.(Gly140Arg)	PV (PP3 + PM2 + PM3 + PM1_Supporting + PP4)	< 0.01%	0%	0.9879	−1.16, −2.07	[16, 26–28]
	c.1318 T > C	p.(Trp440Arg)	LPV (PM2 + PM3 + PM5^b^ + PP3 + PP4)	0%	0%	0.924	−1.63, −2.56	[29]
	c.1500 + 1G > A	Aberrant splicing	LPV (PVS1_Strong + PM2 + PM3 + PS1_Supporting)	0%	0%	NA	NA	[1]
	c.1820del	p.(Leu607HisfsTer4)	LPV (PVS1_Strong + PM2 + PM3 + PP4)	< 0.01%	0%	NA	NA	[1, 30]
	c.1976A > C	p.(Gln659Pro)	VUS (PM2 + PM3 + PP4)	0%	0.03%	0.5189	−0.07, −1.92	[1]
	c.2017 T > C	p.(Cys673Arg)	LPV (PP3 + PM2 + PM5^c^ + PM3 + PP4)	< 0.01%	0.12%	0.9689	−0.82, −2.16	[1, 28, 30–32]
*SLC7A9*	c.404A > G	p.(Tyr135Cys)	VUS (PM2 + PP3)	0%	0%	0.8719	−2.12, −2.89	Novel
	c.829G > A	p.(Val277Met)	VUS (PS4_Moderate + PP4)	0.06%	0.64%	0.629	−0.60, −0.15	[33, 34]
	c.988G > A	p.(Val330Met)	VUS (PM2 + PS4_Supporting + PM1_Supporting)	0.01%	0%	0.7599	−0.71, −0.55	[16, 35]
	c.1060G > A	p.(Ala354Thr)	LPV (PM2 + PS4_Moderate + PS3_Moderate + PP3 + PM1_Supporting)	< 0.01%	0%	0.856	−0.70, −0.02	[9, 16, 28, 36–42]
	c.1305G > A	p.(Trp435Ter)	PV (PVS1 + PM2 + PP4 + PS4_Supporting)	0%	0%	NA	NA	[42]
	c.1445C > T	p.(Pro482Leu)	LPV (PM2 + PM5 + PS4_Moderate + PP4)	< 0.01%	0%	0.632	−0.19, −1.80	[1, 27, 31, 38, 42–47]

*PV* pathogenic variant, *LPV* likely pathogenic variant, *VUS* variant of uncertain significance, *NA* not applicable

^a^The two values indicate the ΔΔG^Stability^ (kcal/mol) when one dimer is affected and when both chains of the dimer are affected, respectively

^b^The rule PM5 was assigned as *SLC3A1* p.(Trp440Cys) was identified in homozygous state from a cystinuria patient in Zhan et al. [48]

^c^The rule PM5 was assigned as *SLC3A1* p.(Cys673Trp) was identified along with *SLC3A1* c.766-2A > G from a cystinuria patient in Bisceglia et al. [32]

**Table 5:** Application of the PM3 criterion for variants identified in *SLC3A1*

Gene	Variant	Predicted effect	PM3 evidence strength (total points)	PM3 points	Reported variants and phase	References
*SLC3A1*	c.223C > T	p.(Gln75Ter)	NA^a^			Novel
	c.418G > A	p.(Gly140Arg)	PM3 (1)	0.5	c.418G > A(;)1515_1516del	[26]
				0.5	c.418G > A(;)1084G > A	[28]
	c.1318 T > C	p.(Trp440Arg)	PM3 (1)	0.5	c.223C > T(;)1318 T > C^a^	This report
				0.5	c.1318 T > C (Hom)	[29]
	c.1500 + 1G > A	Aberrant splicing	PM3 (1)	0.5	c.1500 + 1G > A (Hom)	This report
				0.5	c.46A > T(;)c.1500 + 1G > A	[1]
	c.1820del	p.(Leu607HisfsTer4)	PM3 (1.5)	1.0	c.1820del (Hom)	This report and [1, 30, 31]
				0.5	c.647C > T(;)1820del	[1]
				0.0	c.1820del(;)1976A > C	[1]
	c.1976A > C	p.(Gln659Pro)	PM3 (1.0)	0.5	c.1976A > C(;)2017 T > C	This report
				0.5	c.1820del(;)1976A > C	[1]
	c.2017 T > C	p.(Cys673Arg)	PM3 (1.5)	0.5	c.458 T > C(;)2017 T > C	[31]
				0.5	c.647C > T(;)2017 T > C	[1]
				0.5	c.1501–18_1512del(;)2017 T > C	[28]

*NA* not applicable, *Hom* homozygous

^a^As c.1318 T > C is classified as a likely pathogenic variant with the help of PM3 assigned considering the coincidence with c.223C > T, the PM3 criterion is not applicable for c.223C > T

**Table 6:** Predicted protein stability change in cases with compound heterozygous variants assumed to be in *trans*

Case	*SLC3A1*	*SLC7A9*	DynaMut2
2	ND	c.404A > G(;)988G > A	−1.21
3	c.223C > T(;)1318 T > C	ND	NA
5	c.418G > A(;)1976A > C	ND	−2.38
6	ND	c.1060G > A(;)829G > A	−1.12
8	c.1976A > C(;)2017 T > C	NT	−2.09

*ND* not detected, *NT* not tested, *NA* not applicable

## Discussion

Since the discovery of the underlying genetic defects in cystinuria, a number of variants in *SLC3A1* and *SLC7A9* have been reported to cause cystinuria. However, interpretation of cystinuria-related variants is still challenging for several reasons. Molecular variants in the b^0,+^ transport system are known to cause cystinuria by various functional defects including trafficking, protein folding, protein expression, and amino acid transport [24]. While the current guidelines for variant interpretation embrace the decrease in protein expression by assigning evidence for null variants with consideration of the nonsense-mediated decay mechanism, the degradation of a translated protein due to instability is still underappreciated.

In this study, we reviewed the *SLC3A1* and *SLC7A9* variants identified from cystinuria patients of our institute, which revealed novel variants not reported to date as well as reclassified a previously reported variant of uncertain significance as a likely pathogenic variant. In addition, the results of our evaluation suggest the utility of REVEL in predicting the functional change of the transporter system. However, while REVEL demonstrated different scores for *SLC7A9* variants with mild functional defect (score < 0.6) and severe functional loss (score ≥ 0.8), we were unable to establish a definitive cutoff for REVEL due to limited functional studies with no variants with the degree of functional change elucidated falling into the grey zone (0.6 ≤ score < 0.8). In addition, there were no available functional studies for the *SLC3A1* variants. The protein stability change estimated with DynaMut2 correlated well with the decrease in protein expression level resulting from the p.(Gly105Arg) variant of *SLC7A9*. Among the variants of uncertain significance identified in our study were two variants (*SLC3A1* p.(Gln659Pro) and *SLC7A9* p.(Tyr135Cys)) predicted by DynaMut2 to result in a highly destabilizing protein. We suspect that these variants favor pathogenicity despite insufficient evidence according to the current guidelines. Although DynaMut2 correlated well with the significantly reduced protein expression of *SLC7A9* p.(Gly105Arg), interpretation of the predicted protein instability should be done cautiously since most protein stability prediction tools including DynaMut2 tend to have a bias toward destabilization [25]. As none of the patients with two different variants had a familial history of urolithiasis, the most likely scenario is that both variants in a patient predispose to cystinuria and each of the variants was inherited from a different parent.

Although in silico tools serve as a powerful resource in predicting the pathogenicity of a variant, there are times when the evidence is not sufficient to classify a variant as pathogenic or likely pathogenic despite the variant being highly suspicious as the cause of cystinuria considering other information and the criteria assigned. While some variants are still classified as a variant of uncertain significance despite being highly suspected for pathogenicity, this report adds to the literature and will hopefully be utilized as further evidence for assigning any pathogenic criteria and aid further reports of these variants. Criteria such as PS4 and PM3 could be applied with different weight depending on the number of reported cases.

The diversity of cystinuria-related variants has been reported to be different depending on the ethnic group. While the variant spectrum is unknown regarding the Korean population, it is of note that the variants identified in this study showed a different pattern from a previous Korean publication that included seven cystinuria patients with genetic studies conducted [1].

There are several limitations to our study. First, three patients in our study lacked *SLC7A9* sequencing results as the previous workflow of our institute was to order *SLC7A9* sequencing if no variants were identified from *SLC3A1*. However, two patients carried homozygous likely pathogenic variants in *SLC3A1,* and one patient carried a pathogenic variant and a variant of uncertain significance in *SLC3A1*. As the variant of uncertain significance was deficient of one supporting evidence from being classified as a likely pathogenic variant, we suggest that cystinuria in all three cases could be explained with the variants identified in *SLC3A1*. Second, genetic tests of family members were not carried out. As the designation of phase in patients harboring two different mutations would allow applying stronger evidence in the PM3 criterion, the lack of familial data limits the variant interpretation. Moreover, family members carrying one of the two variants identified in the proband would provide a more sophisticated genotype–phenotype correlation. Third, urinary amino acid levels were measured during the course of treatment. Since the measured concentrations are from different stages of the disease, the data could not be used for genotype–phenotype correlation of the disease.

In summary, we report ten cystinuria cases and the interpretation of variants identified in *SLC3A1* and *SLC7A9*, which included a novel variant in each gene. Our study implies the significance of reporting variants and literature review in determining the pathogenicity of a variant. Moreover, we suggest the potential role of protein stability in predicting loss of function caused by a decrease in protein expression. Hence, this study would benefit future variant interpretation by serving as a list of clinical cases as well as suggesting the approach of utilizing protein instability.

## Data Availability

The data of this study are available upon request from the corresponding author.

## References

[1] Kim JH, Park E, Hyun HS, Lee BH, Kim GH, Lee JH, Park YS, Kang HG, Ha IS, Cheong HI (2017). Genotype and phenotype analysis in pediatric patients with cystinuria. J Korean Med Sci.

[2] Dello Strologo L, Pras E, Pontesilli C, Beccia E, Ricci-Barbini V, de Sanctis L, Ponzone A, Gallucci M, Bisceglia L, Zelante L, Jimenez-Vidal M, Font M, Zorzano A, Rousaud F, Nunes V, Gasparini P, Palacin M, Rizzoni G (2002). Comparison between SLC3A1 and SLC7A9 cystinuria patients and carriers: a need for a new classification. J Am Soc Nephrol.

[3] Broer S, Wagner CA (2002). Structure-function relationships of heterodimeric amino acid transporters. Cell Biochem Biophys.

[4] Wu D, Grund TN, Welsch S, Mills DJ, Michel M, Safarian S, Michel H (2020). Structural basis for amino acid exchange by a human heteromeric amino acid transporter. Proc Natl Acad Sci USA.

[5] Palacin M, Errasti-Murugarren E, Rosell A (2016). Heteromeric amino acid transporters. In search of the molecular bases of transport cycle mechanisms. Biochem Soc Trans.

[6] Wagner CA, Lang F, Broer S (2001). Function and structure of heterodimeric amino acid transporters. Am J Physiol Cell Physiol.

[7] Eggermann T, Venghaus A, Zerres K (2012). Cystinuria: an inborn cause of urolithiasis. Orphanet J Rare Dis.

[8] Lee SY, Kim JW (2000). Physical analysis of urinary stone using fourier transform infrared spectroscopy. Ann Lab Med.

[9] Font MA, Feliubadalo L, Estivill X, Nunes V, Golomb E, Kreiss Y, Pras E, Bisceglia L, d'Adamo AP, Zelante L, Gasparini P, Bassi MT, George AL, Manzoni M, Riboni M, Ballabio A, Borsani G, Reig N, Fernandez E, Zorzano A, Bertran J, Palacin M, International Cystinuria C (2001). Functional analysis of mutations in SLC7A9, and genotype-phenotype correlation in non-Type I cystinuria. Hum Mol Genet.

[10] Ioannidis NM, Rothstein JH, Pejaver V, Middha S, McDonnell SK, Baheti S, Musolf A, Li Q, Holzinger E, Karyadi D, Cannon-Albright LA, Teerlink CC, Stanford JL, Isaacs WB, Xu J, Cooney KA, Lange EM, Schleutker J, Carpten JD, Powell IJ, Cussenot O, Cancel-Tassin G, Giles GG, MacInnis RJ, Maier C, Hsieh CL, Wiklund F, Catalona WJ, Foulkes WD, Mandal D, Eeles RA, Kote-Jarai Z, Bustamante CD, Schaid DJ, Hastie T, Ostrander EA, Bailey-Wilson JE, Radivojac P, Thibodeau SN, Whittemore AS, Sieh W (2016). REVEL: an ensemble method for predicting the pathogenicity of rare missense variants. Am J Hum Genet.

[11] Rodrigues CHM, Pires DEV, Ascher DB (2021). DynaMut2: Assessing changes in stability and flexibility upon single and multiple point missense mutations. Protein Sci.

[12] Ellard S, Baple EL, Berry I, Forrester N, Turnbull C, Owens MM, Eccles DM, Abbs SJ, Scott R, Deans ZC, Lester T, Jo C, Newman WG, McMullan D (2019) ACGS best practice guidelines for variant classification. https://www.acgs.uk.com/media/11285/uk-practice-guidelines-for-variant-classification-2019-v1-0-3.pdf. Accessed 23 Jan 2023

[13] Richards S, Aziz N, Bale S, Bick D, Das S, Gastier-Foster J, Grody WW, Hegde M, Lyon E, Spector E, Voelkerding K, Rehm HL, Committee ALQA (2015). Standards and guidelines for the interpretation of sequence variants: a joint consensus recommendation of the American College of Medical Genetics and Genomics and the Association for Molecular Pathology. Genet Med.

[14] SVI Recommendation for Absence/Rarity (PM2)—Version 1.0. Available from: https://clinicalgenome.org/site/assets/files/5182/pm2_-_svi_recommendation_-_approved_sept2020.pdf. Accessed 23 Jan 2023

[15] SVI Recommendation for in trans Criterion (PM3)—Version 1.0. Available from: https://clinicalgenome.org/site/assets/files/3717/svi_proposal_for_pm3_criterion_-_version_1.pdf. Accessed 23 Jan 2023

[16] Martell HJ, Wong KA, Martin JF, Kassam Z, Thomas K, Wass MN (2017). Associating mutations causing cystinuria with disease severity with the aim of providing precision medicine. BMC Genom.

[17] Stenson PD, Ball EV, Mort M, Phillips AD, Shiel JA, Thomas NS, Abeysinghe S, Krawczak M, Cooper DN (2003). Human Gene Mutation Database (HGMD): 2003 update. Hum Mutat.

[18] Chunn LM, Nefcy DC, Scouten RW, Tarpey RP, Chauhan G, Lim MS, Elenitoba-Johnson KSJ, Schwartz SA, Kiel MJ (2020). Mastermind: a comprehensive genomic association search engine for empirical evidence curation and genetic variant interpretation. Front Genet.

[19] Karczewski KJ, Francioli LC, Tiao G, Cummings BB, Alföldi J, Wang Q, Collins RL, Laricchia KM, Ganna A, Birnbaum DP, Gauthier LD, Brand H, Solomonson M, Watts NA, Rhodes D, Singer-Berk M, England EM, Seaby EG, Kosmicki JA, Walters RK, Tashman K, Farjoun Y, Banks E, Poterba T, Wang A, Seed C, Whiffin N, Chong JX, Samocha KE, Pierce-Hoffman E, Zappala Z, O’Donnell-Luria AH, Minikel EV, Weisburd B, Lek M, Ware JS, Vittal C, Armean IM, Bergelson L, Cibulskis K, Connolly KM, Covarrubias M, Donnelly S, Ferriera S, Gabriel S, Gentry J, Gupta N, Jeandet T, Kaplan D, Llanwarne C, Munshi R, Novod S, Petrillo N, Roazen D, Ruano-Rubio V, Saltzman A, Schleicher M, Soto J, Tibbetts K, Tolonen C, Wade G, Talkowski ME, Aguilar Salinas CA, Ahmad T, Albert CM, Ardissino D, Atzmon G, Barnard J, Beaugerie L, Benjamin EJ, Boehnke M, Bonnycastle LL, Bottinger EP, Bowden DW, Bown MJ, Chambers JC, Chan JC, Chasman D, Cho J, Chung MK, Cohen B, Correa A, Dabelea D, Daly MJ, Darbar D, Duggirala R, Dupuis J, Ellinor PT, Elosua R, Erdmann J, Esko T, Färkkilä M, Florez J, Franke A, Getz G, Glaser B, Glatt SJ, Goldstein D, Gonzalez C, Groop L, Haiman C, Hanis C, Harms M, Hiltunen M, Holi MM, Hultman CM, Kallela M, Kaprio J, Kathiresan S, Kim B-J, Kim YJ, Kirov G, Kooner J, Koskinen S, Krumholz HM, Kugathasan S, Kwak SH, Laakso M, Lehtimäki T, Loos RJF, Lubitz SA, Ma RCW, MacArthur DG, Marrugat J, Mattila KM, McCarroll S, McCarthy MI, McGovern D, McPherson R, Meigs JB, Melander O, Metspalu A, Neale BM, Nilsson PM, O’Donovan MC, Ongur D, Orozco L, Owen MJ, Palmer CNA, Palotie A, Park KS, Pato C, Pulver AE, Rahman N, Remes AM, Rioux JD, Ripatti S, Roden DM, Saleheen D, Salomaa V, Samani NJ, Scharf J, Schunkert H, Shoemaker MB, Sklar P, Soininen H, Sokol H, Spector T, Sullivan PF, Suvisaari J, Tai ES, Teo YY, Tiinamaija T, Tsuang M, Turner D, Tusie-Luna T, Vartiainen E, Vawter MP, Ware JS, Watkins H, Weersma RK, Wessman M, Wilson JG, Xavier RJ, Neale BM, Daly MJ, MacArthur DG, Genome Aggregation Database C (2020). The mutational constraint spectrum quantified from variation in 141,456 humans. Nature.

[20] Jung KS, Hong KW, Jo HY, Choi J, Ban HJ, Cho SB, Chung M (2020) KRGDB: the large-scale variant database of 1722 Koreans based on whole genome sequencing. Database (Oxford) 2020:baz146 10.1093/database/baaa030 PMC7190023 32348452

[21] Jaganathan K, Kyriazopoulou Panagiotopoulou S, McRae JF, Darbandi SF, Knowles D, Li YI, Kosmicki JA, Arbelaez J, Cui W, Schwartz GB, Chow ED, Kanterakis E, Gao H, Kia A, Batzoglou S, Sanders SJ, Farh KK (2019). Predicting splicing from primary sequence with deep learning. Cell.

[22] Ittisoponpisan S, Islam SA, Khanna T, Alhuzimi E, David A, Sternberg MJE (2019). Can predicted protein 3D structures provide reliable insights into whether missense variants are disease associated?. J Mol Biol.

[23] Yan R, Li Y, Shi Y, Zhou J, Lei J, Huang J, Zhou Q (2020). Cryo-EM structure of the human heteromeric amino acid transporter b(0,+)AT-rBAT. Sci Adv.

[24] Chillaron J, Font-Llitjos M, Fort J, Zorzano A, Goldfarb DS, Nunes V, Palacin M (2010). Pathophysiology and treatment of cystinuria. Nat Rev Nephrol.

[25] Pancotti C, Benevenuta S, Birolo G, Alberini V, Repetto V, Sanavia T, Capriotti E, Fariselli P (2022). Predicting protein stability changes upon single-point mutation: a thorough comparison of the available tools on a new dataset. Brief Bioinform.

[26] Skopková Z, Hrabincová E, Stástná S, Kozák L, Adam T (2005). Molecular genetic analysis of SLC3A1 and SLC7A9 genes in Czech and Slovak cystinuric patients. Ann Hum Genet.

[27] Wong KA, Mein R, Wass M, Flinter F, Pardy C, Bultitude M, Thomas K (2015). The genetic diversity of cystinuria in a UK population of patients. BJU Int.

[28] Tostivint I, Royer N, Nicolas M, Bourillon A, Czerkiewicz I, Becker PH, Muller F, Benoist JF (2017). Spectrum of mutations in cystinuria patients presenting with prenatal hyperechoic colon. Clin Genet.

[29] Gaildrat P, Lebbah S, Tebani A, Sudrie-Arnaud B, Tostivint I, Bollee G, Tubeuf H, Charles T, Bertholet-Thomas A, Goldenberg A, Barbey F, Martins A, Saugier-Veber P, Frebourg T, Knebelmann B, Bekri S (2017). Clinical and molecular characterization of cystinuria in a French cohort: relevance of assessing large-scale rearrangements and splicing variants. Mol Genet Genomic Med.

[30] Egoshi KI, Akakura K, Kodama T, Ito H (2000). Identification of five novel SLC3A1 (rBAT) gene mutations in Japanese cystinuria. Kidney Int.

[31] Shigeta Y, Kanai Y, Chairoungdua A, Ahmed N, Sakamoto S, Matsuo H, Kim DK, Fujimura M, Anzai N, Mizoguchi K, Ueda T, Akakura K, Ichikawa T, Ito H, Endou H (2006). A novel missense mutation of SLC7A9 frequent in Japanese cystinuria cases affecting the C-terminus of the transporter. Kidney Int.

[32] Bisceglia L, Purroy J, Jimenez-Vidal M, d'Adamo AP, Rousaud F, Beccia E, Penza R, Rizzoni G, Gallucci M, Palacin M, Gasparini P, Nunes V, Zelante L (2001). Cystinuria type I: identification of eight new mutations in SLC3A1. Kidney Int.

[33] Shen L, Cong X, Zhang X, Wang N, Zhou P, Xu Y, Zhu Q, Gu X (2017). Clinical and genetic characterization of Chinese pediatric cystine stone patients. J Pediatr Urol.

[34] Sun Y, Man J, Wan Y, Pan G, Du L, Li L, Yang Y, Qiu L, Gao Q, Dan H, Mao L, Cheng Z, Fan C, Yu J, Lin M, Kristiansen K, Shen Y, Wei X (2018). Targeted next-generation sequencing as a comprehensive test for Mendelian diseases: a cohort diagnostic study. Sci Rep.

[35] Harnevik L, Fjellstedt E, Molbaek A, Denneberg T, Soderkvist P (2003). Mutation analysis of SLC7A9 in cystinuria patients in Sweden. Genet Test.

[36] Reig N, Chillaron J, Bartoccioni P, Fernandez E, Bendahan A, Zorzano A, Kanner B, Palacin M, Bertran J (2002). The light subunit of system b(o,+) is fully functional in the absence of the heavy subunit. EMBO J.

[37] Leclerc D, Boutros M, Suh D, Wu Q, Palacin M, Ellis JR, Goodyer P, Rozen R (2002). SLC7A9 mutations in all three cystinuria subtypes. Kidney Int.

[38] Bisceglia L, Fischetti L, Bonis PD, Palumbo O, Augello B, Stanziale P, Carella M, Zelante L (2010). Large rearrangements detected by MLPA, point mutations, and survey of the frequency of mutations within the SLC3A1 and SLC7A9 genes in a cohort of 172 cystinuric Italian patients. Mol Genet Metab.

[39] Abe Y, Sakamoto S, Morimoto E, Watanabe Y, Nagahara K, Mikawa T, Watanabe S, Itabashi K (2014) Persistent leukocyturia was a clue to diagnosis of cystinuria in a female patient. Glob Pediatr Health 1:2333794X14551275 10.1177/2333794X14551275 PMC4804682 27335907

[40] Rhodes HL, Yarram-Smith L, Rice SJ, Tabaksert A, Edwards N, Hartley A, Woodward MN, Smithson SL, Tomson C, Welsh GI, Williams M, Thwaites DT, Sayer JA, Coward RJ (2015). Clinical and genetic analysis of patients with cystinuria in the United Kingdom. Clin J Am Soc Nephrol.

[41] Alghamdi M, Alhasan KA, Taha Elawad A, Salim S, Abdelhakim M, Nashabat M, Raina R, Kari J, Alfadhel M (2020). Diversity of phenotype and genetic etiology of 23 cystinuria saudi patients: a retrospective study. Front Pediatr.

[42] Tkaczyk M, Gadomska-Prokop K, Zaluska-Lesniewska I, Musial K, Zawadzki J, Jobs K, Porowski T, Rogowska-Kalisz A, Jander A, Kirolos M, Halinski A, Krzemien A, Sobieszczanska-Drozdziel A, Zachwieja K, Beck BB, Sikora P, Zaniew M (2021). Clinical profile of a Polish cohort of children and young adults with cystinuria. Ren Fail.

[43] Lotan D, Yoskovitz G, Bisceglia L, Gerad L, Reznik-Wolf H, Pras E (2007). A combined approach to the molecular analysis of cystinuria: from urinalysis to sequencing via genotyping. Isr Med Assoc J.

[44] Sakamoto S, Chairoungdua A, Nagamori S, Wiriyasermkul P, Promchan K, Tanaka H, Kimura T, Ueda T, Fujimura M, Shigeta Y, Naya Y, Akakura K, Ito H, Endou H, Ichikawa T, Kanai Y (2009). A novel role of the C-terminus of b 0,+ AT in the ER-Golgi trafficking of the rBAT-b 0,+ AT heterodimeric amino acid transporter. Biochem J.

[45] Tohge R, Sakamoto S, Takahashi M (2016). A case of cystinuria presenting with cerebellar ataxia and dementia. Pract Neurol.

[46] Okada T, Taguchi K, Kato T, Sakamoto S, Ichikawa T, Yasui T (2021). Efficacy of transurethral cystolithotripsy assisted by percutaneous evacuation and the benefit of genetic analysis in a pediatric cystinuria patient with a large bladder stone. Urol Case Rep.

[47] Ikeyama S, Kanda S, Sakamoto S, Sakoda A, Miura K, Yoneda R, Nogi A, Ariji S, Shimoda M, Ono M, Kanda S, Yokoyama S, Takahashi K, Yokoyama Y, Hattori M (2022). A case of early onset cystinuria in a 4-month-old girl. CEN Case Rep.

[48] Zhan R, Ge Y, Liu Y, Zhao Z, Wang W (2022). Genetic and clinical analysis of Chinese pediatric patients with cystinuria. Urolithiasis.

